# Seasonal diets supersede host species in shaping the distal gut microbiota of Yaks and Tibetan sheep

**DOI:** 10.1038/s41598-021-99351-4

**Published:** 2021-11-19

**Authors:** Xiaojuan Wei, Zhen Dong, Fusheng Cheng, Hongmei Shi, Xuzheng Zhou, Bing Li, Ling Wang, Weiwei Wang, Jiyu Zhang

**Affiliations:** 1 Key Lab of New Animal Drug Project, Lanzhou, 730050 Gansu Province People’s Republic of China; 2 Key Lab of Veterinary Pharmaceutical Development, Ministry of Agriculture, Lanzhou, 730050 People’s Republic of China; 3 Lanzhou Institute of Husbandry and Pharmaceutical Science of Chinese Academy of Agricultural Sciences, Lanzhou, People’s Republic of China; 4 Gannan Tibetan Autonomous Prefecture Institute of Animal Husbandry Science, Hezuo, People’s Republic of China

**Keywords:** Biodiversity, Applied microbiology

## Abstract

Yaks and Tibetan sheep are important and renowned livestock of the Qinghai-Tibetan Plateau (QTP). Both host genetics and environmental factors can shape the composition of gut microbiota, however, there is still no consensus on which is the more dominant factor. To investigate the influence of hosts and seasons on the gut microbiome diversity component, we collected fecal samples from yaks and Tibetan sheep across different seasons (summer and winter), during which they consumed different diets. Using 16S rRNA sequencing, principal component analysis (PCoA) data showed that PCo1 explained 57.4% of the observed variance (P = 0.001) and clearly divided winter samples from summer ones, while PCo2 explained 7.1% of observed variance (P = 0.001) and mainly highlighted differences in host species. Cluster analysis data revealed that the gut microbiota composition displayed a convergence caused by season and not by genetics. Further, we profiled the gut microbial community and found that the more dominant genera in yak and Tibetan sheep microbiota were influenced by seasonal diets factors rather than genetics. This study therefore indicated that seasonal diet can trump host genetics even at higher taxonomic levels, thus providing a cautionary note for the breeding and management of these two species.

## Introduction

As one of the largest natural alpine grasslands in the world, the Qinghai-Tibetan Plateau (QTP) is an ideal location for ecological animal husbandry with an environment that scarcely changes and is rarely disturbed by humans. The yak (*Bos grunniens*) and the Tibetan sheep (*Ovis aries*) inhabit this region, having adapted to the environment to become the principal livestock animals of the nomadic Tibetan people^1^. Both animals are a source of essential food (milk and meat), transport (mainly the yak), fuel (yak feces), shelter, and clothing (skin and fur), and also fulfill various socio-cultural functions within Tibetan pastoral society. However, the high altitude of the QTP presents an extremely harsh environment for the animals. Due to lack of food, coupled with parasitic diseases results in a high fatality rate and an annual reduction in weight takes place each year as the weight gained while grazing summer fodder is lost due to poorer winter grazing about 20–30%^2^. In order to control parasitic diseases, yaks were administered a single oral gavage of albendazole tablets at doses of 15 mg/kg body weight in January and August, respectively. For ruminant livestock species, the GIT microbiota is indispensable to the nutritive function of the animal^3^.

Regarding these two livestock species, previous studies have compared growth performance, infection risks, and carcass characteristics^4^ and their impact on the environment^5,6^, but few studies have considered the diversity and composition of the gut microbial communities, or the correlation between winter and summer grazing. However, over the last decade, intensive studies have indicated that there are many factors that can shape the composition of mammalian gut microbiota, including host genetics and diet^7,8^. Some reports show that a host’s genotype has a measurable impact on gut microbial properties in both humans^9,10^ and mice^11^. However, other reports indicate that diet can supersede differences in genotype in determining the properties of murine gut microbiota^8^. Most of these studies were performed in one or more of five inbred mouse strains, thus raising the question of how great a phylogenetic distance can be masked through alterations in diet. Recent studies showed that “the microbiomes were co-evolving with the host genomes because of extreme environmental adaptation and the convergent evolution of rumen microbiomes related to energy harvesting persistence in high-altitude ruminants”^12^. While the sample number was small in that study, no comparison of gut microbiota was made between the yak and sheep, just cattle vs. yak and sheep vs. Tibetan sheep. The animals investigated in the current study, the yak and Tibetan sheep, both belong to the same Bovinae family, but to different subfamilies, Bovinae and Caprinae, respectively. In recent years, there were many reports about rumen microbes composition of ruminant^12,13^, however, research on gut microbial contribution to ruminant health and production is still at an early stage. Moreover, the way of collecting ruminal content is invasive and may cause damage to animal’s health. Therefore, considering animal welfare, fecal microbiota is the portion of the final product of digestion, and it could serve as an effective and non-invasive diagnosis of balance in gut health^14^. So, we investigated the composition of gut microbiota during the winter and the summer to examine whether host species genotype or diet had a greater impact in shaping gut microbial populations.

## Results

### Overall profiles of microbial composition

Illumina sequencing yielded a total of 4 363 232 16S rRNA gene sequence raw reads. After filtering for read quality, 3 021 303 valid sequences were clustered into 6784 prokaryotic operational taxonomic units (OTUs) at a 97% sequence identity level. We identified 22 bacterial and 1 archaeal phyla in the investigated samples. Venn diagrams indicated that OTUs were mostly distinguished by season (Fig. S1). *Bacteroidetes* was the most predominant phylum, comprising, on average, 56% of the relative abundance. In winter, both yaks and sheep presented similar relative abundance levels of *Bacteroidetes* in their gut, but in summer, yaks had significantly higher *Bacteroidetes* abundance than sheep (P < 0.001; Fig. S2). That is to say, no significant difference in terms of relative abundance of *Bacteroidetes* was found between winter and summer samples as a whole (P > 0.1). *Firmicutes* was the second most prevalent phylum, averaging 38% of the total prokaryotic community. *Firmicutes* relative abundance did not significantly differ among samples grouped by season or host, but the change in abundance between seasons was different in the two host animals. Variations were much stronger at the family level, especially when samples were compared by season (Fig. S2). *Bacteroidaceae* and *Rikenellaceae* were prevalent in winter, while *Prevotellaceae*, *BS11*, and *S24-7* were abundant during summer. *Fibrobacteraceae* and *Spirochaetaceae* were found mainly in sheep compared with Yak during the winter compared with summer.

### Host and seasonal diets effects on the composition of microbial community and diversity indices

**Figure 1:**
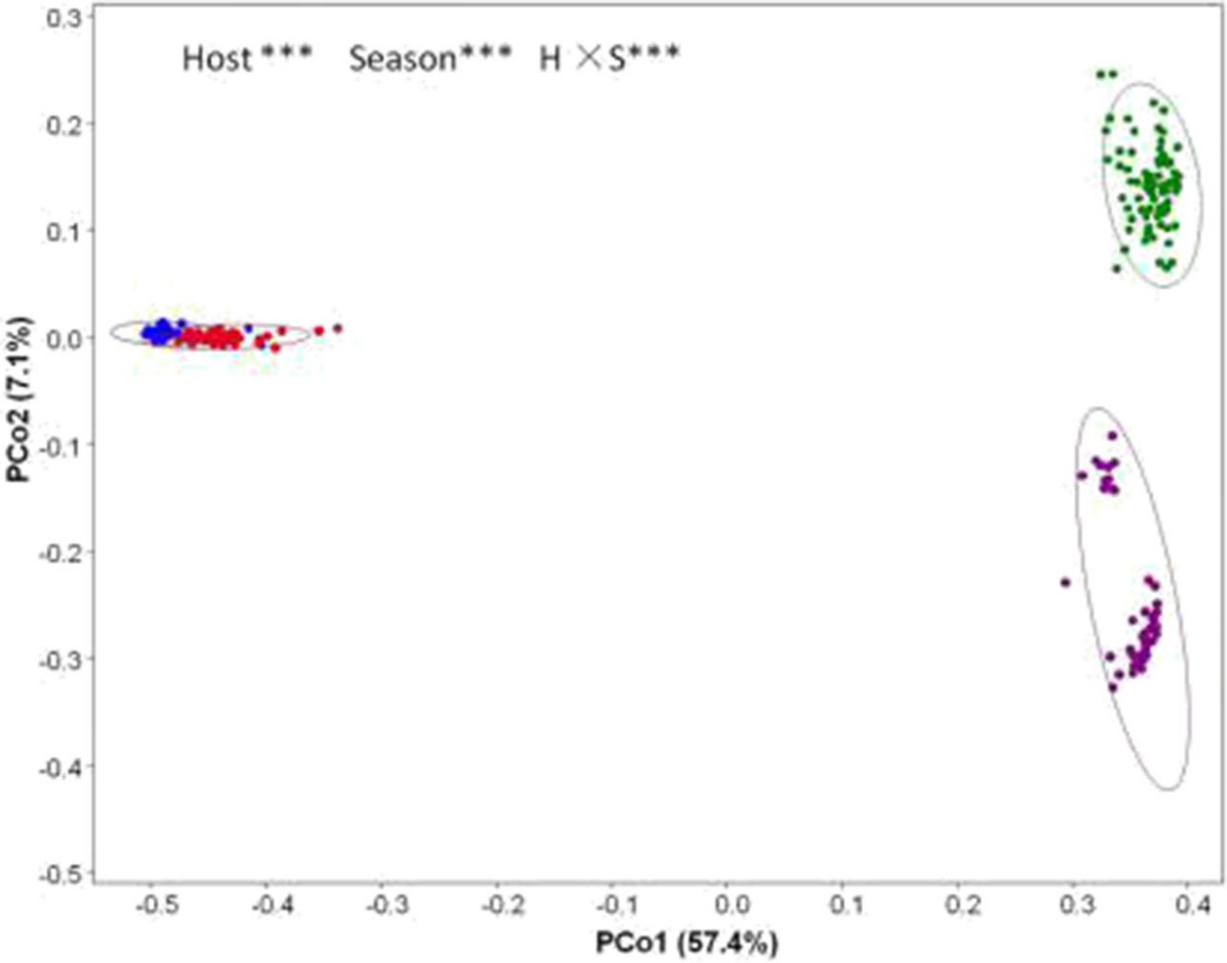
Principal coordinates analysis (PCoA) ordination of the operational taxonomic units (OTUs). Dots indicate one sample and the circles are the 95% ellipses. Colors are as follows: blue = WinY; red = WinS; green = SumY; purple = SumS. Results of PERMANOVA are given in the upper right of each panel: **P < 0.01; ***P < 0.001.

**Figure 2:**
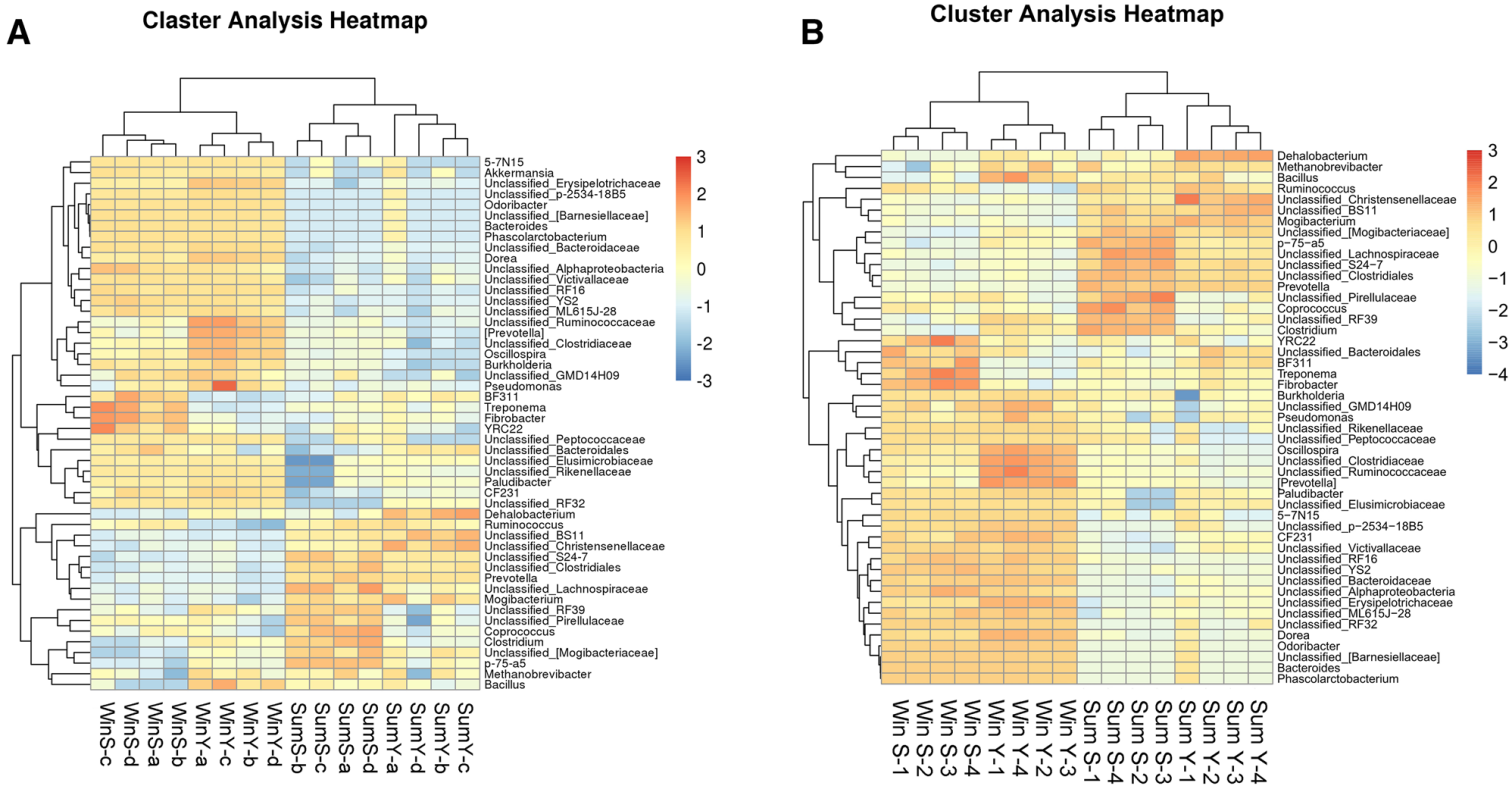
Cluster analysis heatmap of 16 samples randomly selected four from every group (**A**) and 16 sample of averages (**B**). The closer the color is to orange, the more relevant it is. The closer the color is to blue, the less relevant it is.

To investigate the composition of fecal gut microbial communities of the 56 winter yak, 80 summer yak, 43 winter Tibetan sheep, and 47 summer Tibetan sheep, PCoA plots based on 16S rRNA genes were generated to compare the microbial community composition between hosts and seasons (Fig. 1). PCo1 explained 57.4% of the variance observed (P = 0.001) and clearly divided winter samples from summer ones, while PCo2 explained 7.1% of the variance observed (P = 0.001) and mainly highlighted differences in host species (albeit only in summer). Further, in order to make the figure clear and intuitive, we conducted cluster analysis on 16 samples randomly selected per four from every group (Fig. 2A) and 16 sample of averages (Fig. 2B). We calculated the mean for the four groups of samples and then conduct cluster analyses. WinS-a, WinS-b, WinS-c were the averages of 30 samples of winter Tibetan sheep (10 per group), WinS-d were the average of the final 13 samples; WinY-a, WinY-b, WinY-c, and WinY-d were the averages of the 56 samples of winter yak (14 per group); SumS-a, SumS-b, and SumS-c were the averages of 33 samples of summer Tibetan sheep (11 per group), SumS-d included the remaining 14 samples; SumY-a, SumY-b, SumY-c, and SumY-d were the averages of 80 samples of summer yak (20 per group). The data showed that the samples had a convergence caused by seasons and not by genetics.

### Host and seasonal diets effects on microbial community diversity indices

**Figure 3:**
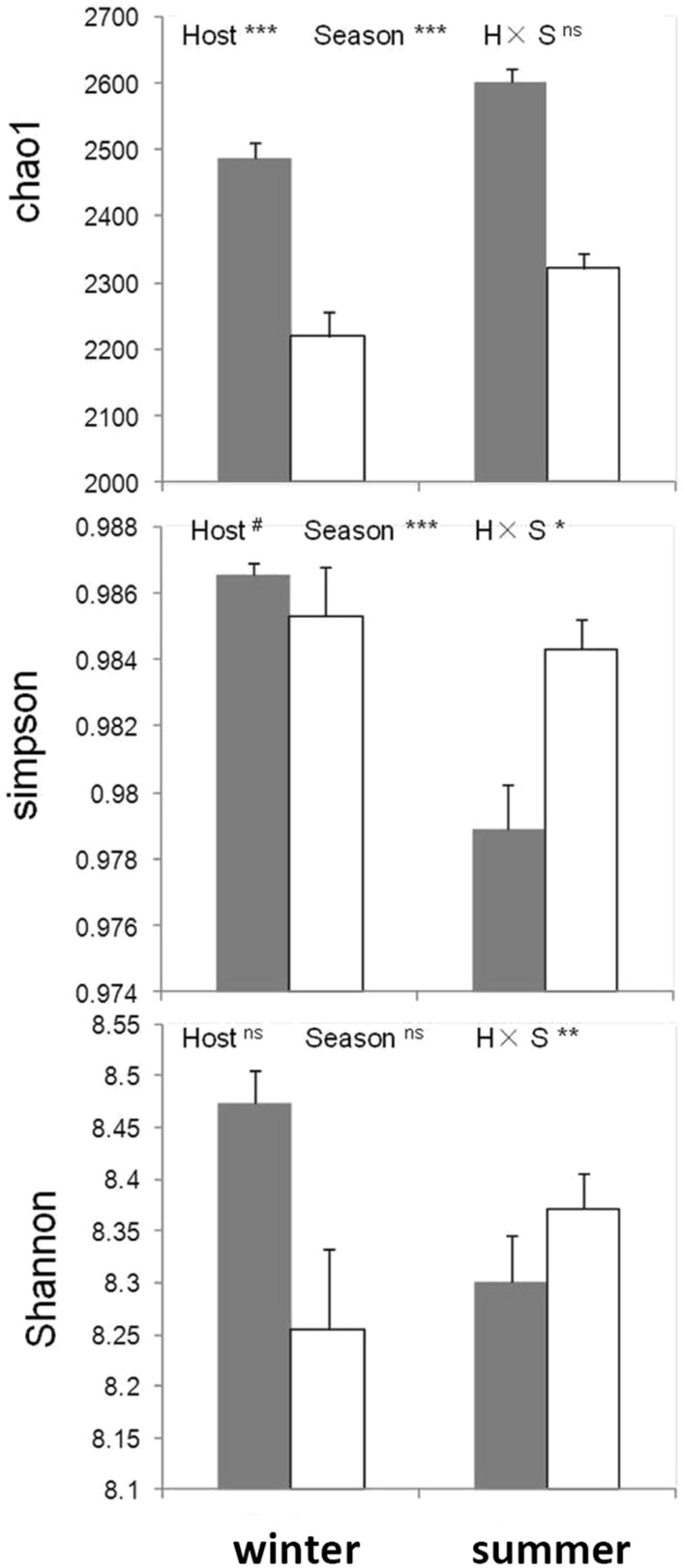
Two-way ANOVA analysis for alpha diversity indices (mean ± SE) with host and season. Gray bars represent the data of yaks and white bars represent Tibetan sheep. *P < 0.05; **P < 0.01; ***P < 0.001; ^#^P < 0.1.

**Figure 4:**
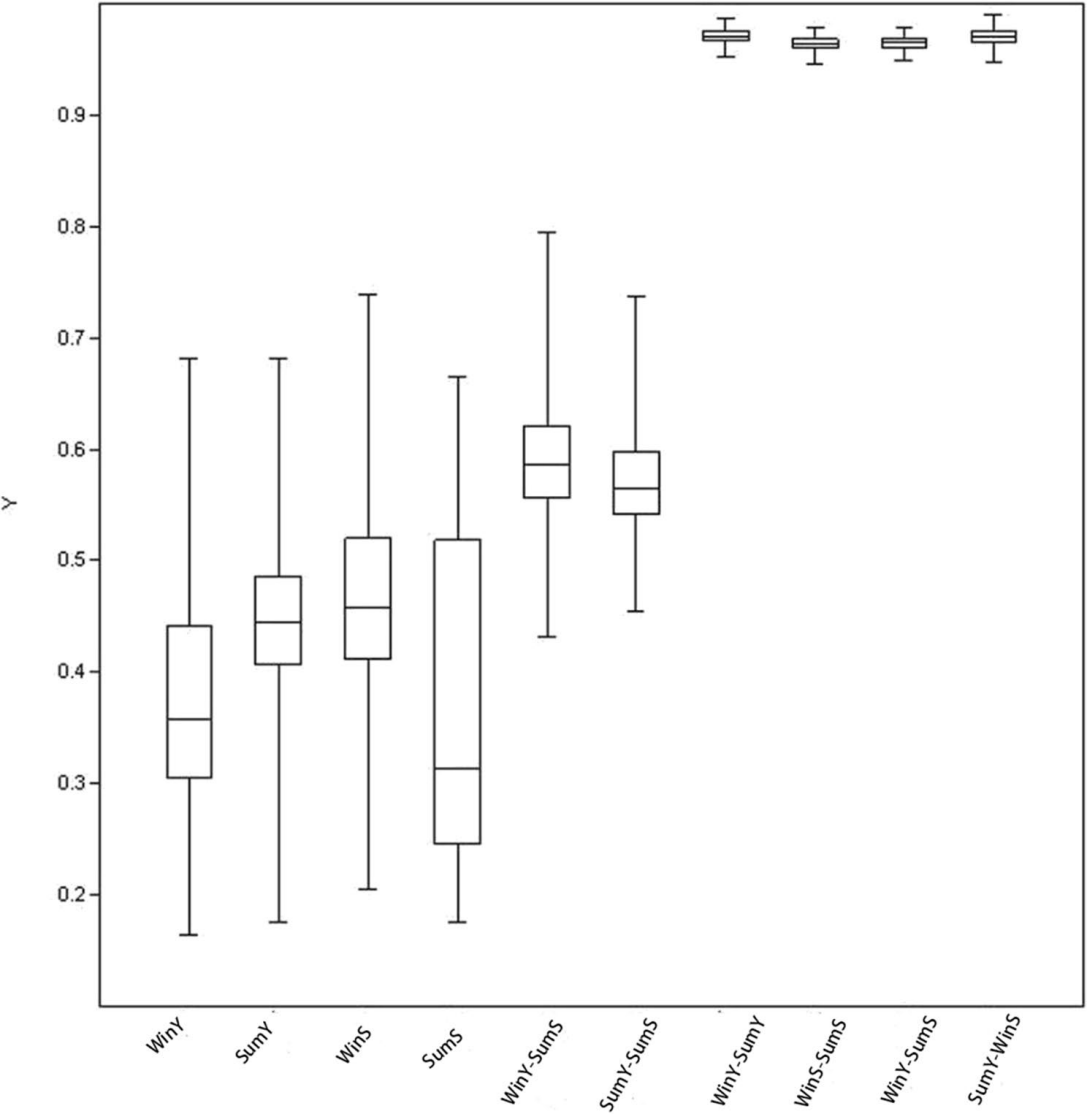
Beta diversity-based Bray–Curtis distances of the samples inside the groups and between the different groups.

Alpha diversity was compared; both host species and season demonstrated highly significant effects on the species richness of gut microbiota (chao1). Yaks always presented greater numbers of prokaryotic species in their gut than Tibetan sheep, with species richness always higher in summer relative to winter (Fig. 3). The host effect was marginally significant to the evenness (Simpson index) but the seasonal diets effect was highly significant. The interaction of these effects was also significant. On the Shannon index, neither host nor season effects were significant, but the interaction of these effects were significant (Fig. 3). Subsequently, we compared the beta diversity; this was much higher between different seasons than between different hosts, with the latter barely higher than beta diversity within the same groups (Fig. 4). All of the above data indicated that season had a greater influence on the composition of gut microbiota than host genetic background, despite the yak and Tibetan sheep belonging to different subfamilies, *Bovinaea* and *Caprinae*, respectively. As for PCoA based on the functional gene composition (Fig. S3), PCo1 explained 70.6% of the variance and PCo2 explained 13% of the variance. However, no groups were clearly distinguished from one another, although PERMANOVA results indicated both a host and season effect, with the interaction between them statistically significant.

### Profiles at a genus level in the gut microbial community of yaks and Tibetan sheep

**Figure 5:**
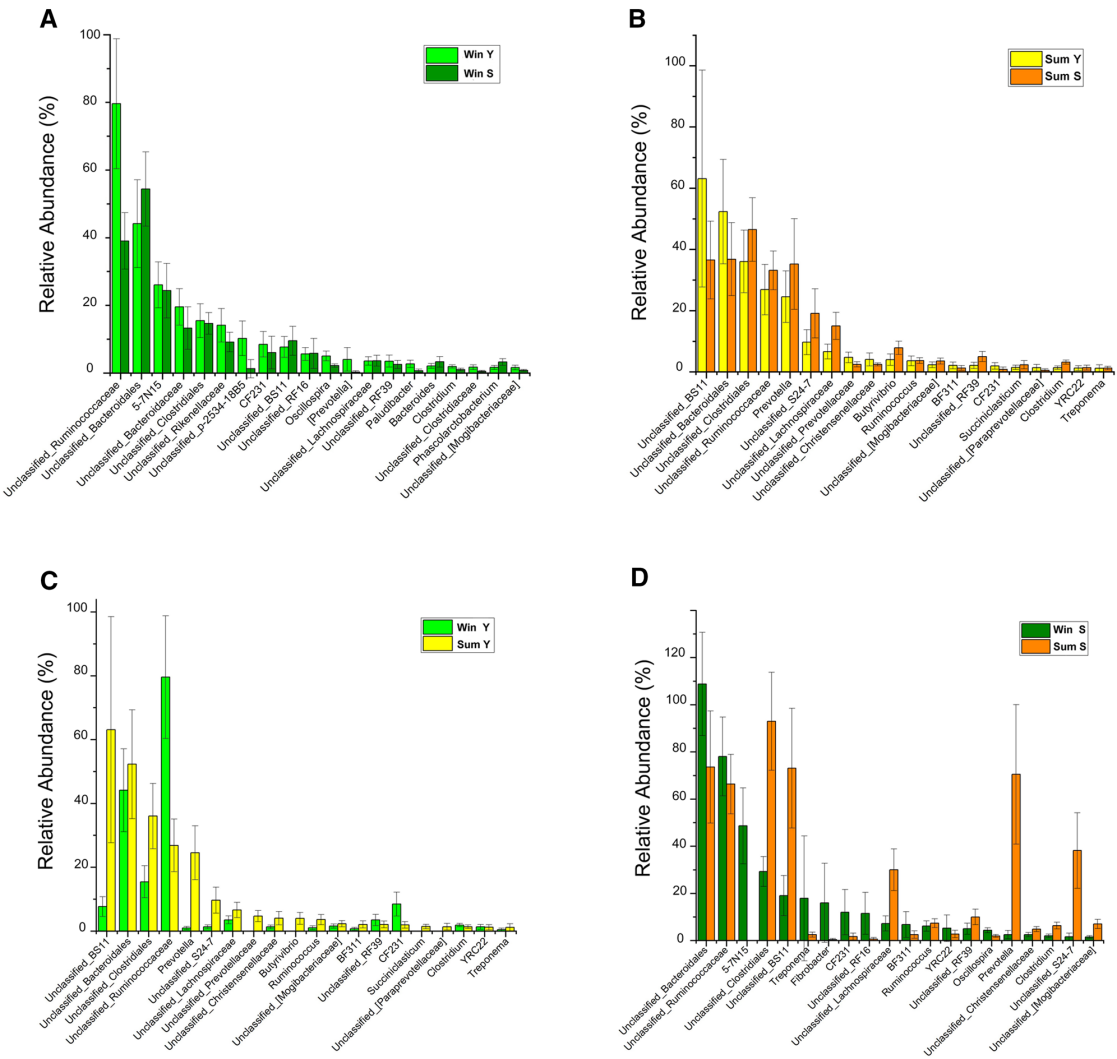
One-way ANOVA to compare the number of genera in groups. Twenty genera that were statistically significant are listed. (**A**) WinS vs. WinY; (**B**) SumS vs. SumY; (**C**) SumY vs. WinY; (**D**) SumS vs. WinS.

In order to compare gut microbiota to observe the influence of season or genetics, we used one-way ANOVA to compare the level of genus in group WinS vs. WinY (Fig. 5A), SumS vs. SumY (Fig. 5B), SumY vs. WinY (Fig. 5C), and SumS vs. WinS (Fig. 5D) and listed the 20 most statistically significant genera. The data showed that 13 main genera were found: *BS11*, *Unclassified Clostridiales*, *Unclassified Ruminococcaceae*, *Prevotella*, *Unclassified Lachnospiraceae, Unclassified Prevotellaceae*, *Unclassified Christensenellaceae*, *Butyrivibrio*, *Ruminococcus*, *CF231*, *Unclassified Mogibacteriaceae*, *Succiniclasticum*, *Unclassified Paraprevotellaceae*. The numbers of these genera were always higher in one season than in another, regardless of species (yak or Tibetan sheep). The amounts of the genera *Unclassified Ruminococcaceae*, *Unclassified_Bacteroidales*, *Unclassified Clostridiales*, *Oscillospira*, *RF39*, and *Clostridium* always changed with the seasons, but not with the host. In different host (yak and Tibetan sheep), the genus of bacterium was remaining constant. So, the dominant genera in gut microbial communities of yak and Tibetan sheep in QTP were influenced by seasonal diets factors rather than genetics, such as *Unclassified Ruminococcaceae* and *Unclassified Clostridiales*.

**Figure 6:**
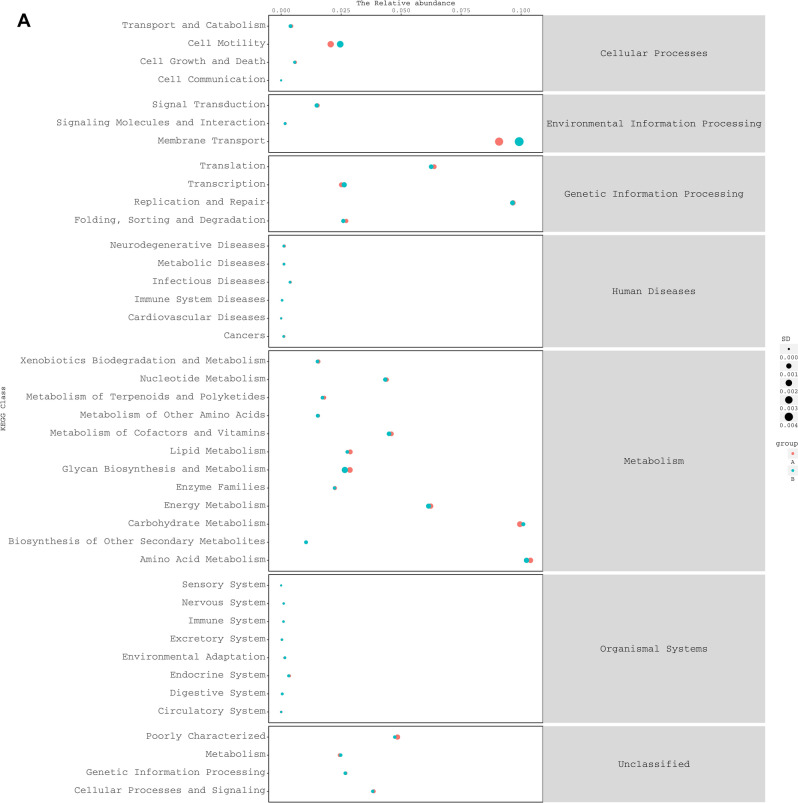

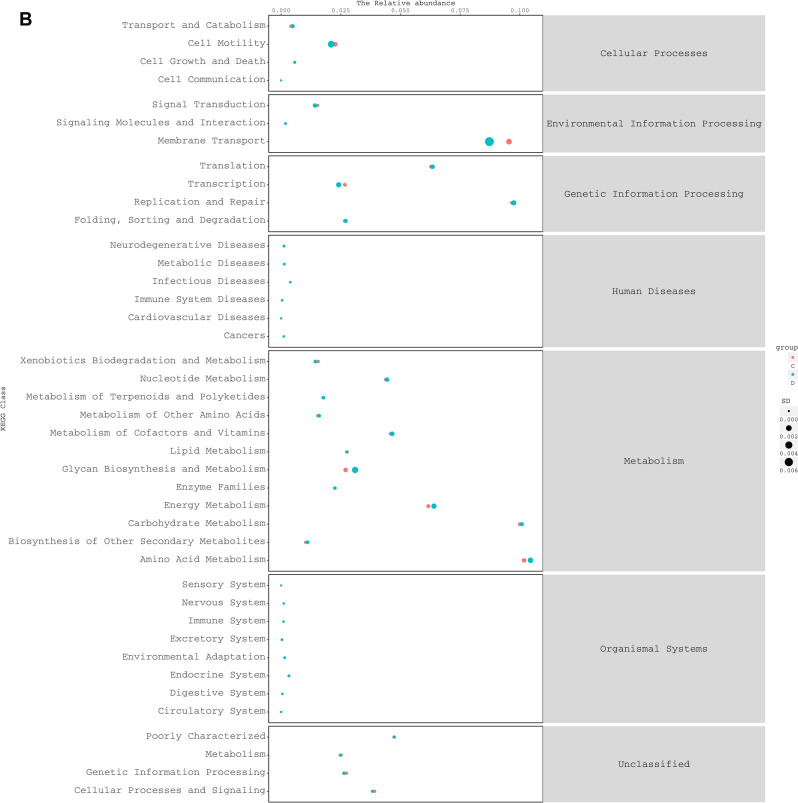
Comparison of predicted KEGG pathways for the fecal bacterial microbiota of Tibetan sheep** (A)**. Comparison of predicted KEGG pathways for the fecal bacterial microbiota of yak **(B)**.

According to PICRUSt analysis, we found 41 KEGG pathways (level 2) (Fig. 6), of which 26 were significantly different between SumS and WinS samples. In these pathways, we pay more attention to substance metabolic pathways, and the top 5 abundant pathways are amino acid metabolism, carbohydrate metabolism, energy metabolism, metabolism of Cofactors and Vitamins, and nucleotide Metabolism. The same result is found in yak. We suggested that the gut flora may enhance the fitness of the animals by maintaining the energy function of the gut flora in the face of nutrient deficiency.

## Discussion

Yak and Tibetan sheep thrive under a co-grazing system on the QTP and/or are fed with the same materials; this offers an excellent opportunity to compare the gut microbiota in different host species which share a similar diet. In addition, the grazing systems on the QTP undergo seasonal diets changes in terms of pasture location and forage composition, especially between winter and summer. This presents a good natural “treatment” which helps vary the diets of the yak and Tibetan sheep populations. In the current study, based on a more substantial sample size than the previous study^1^, we found that diet and environment (represented by seasons winter and summer) superseded host genetics to the family level. That is to say that the gut microbiota of the two animal species showed convergent adaptation to high altitude and harsh environment in QTP, but this convergence had seasonal diets characteristics. These findings may provide a cautionary note for ongoing efforts to link host genetics to gut microbiota composition and function and would provide some food for thought in the breeding of these two livestock groups.

The mammalian gut microbiota is acquired from the environment starting at birth, and its assembly and composition is largely shaped by factors such as age, diet, lifestyle, hygiene, and disease state. Researchers subconsciously believe that host species play a greater role than environmental factors when it comes to shaping gut microbiota, especially when there is a large taxonomical difference between the host species. So far, the vast majority of research have focused on the ruminal ecosystem because the rumen is primary site of feed fermentation^15–17^. It is rare to find studies that directly compare the gut microbiota of different species. However, evidence showed that energetically-important microbial products, including VFA (10–13% of total GIT VFA) are produced in the ruminant distal gut^3^. Hence, it is important to study the composition of distal gut microbiota of ruminants.

In this study, at the phylum level, the gut microbiota composition in both groups of livestock was dominated by *Bacteroidetes* and *Firmicutes*, which was in agreement with previous reports concerning the yak^18^. At the same time our result consistently with other study in dairy cows that two dominated phyla *Bacteroidetes* and *Firmicutes* found in fecal samples in different seasons were abundant^19,20^. *Firmicutes* and *Bacteroidetes* are responsible for digestion of carbohydrates and proteins, Members of *Bacteroidetes* having extremely stronger ability to degrade crystalline cellulose. The previous report showed that intestinal microbiome plays an important role in digestion and absorption of the food, and maintaining animals’ health^21,22^. Intestinal tracts of the ruminants are rich in symbiotic bacteria that helps the body digest plant fibers^23,24^. Glycans are processed by the distal gut microbiota, generating biologically significant short-chain fatty acids (SCFAs, predominantly acetate, butyrate, and propionate), which serve as the principal energy source for colonocytes^25^. Fibers may be involved in the regulation of food intake and energy balance via the SCFA-mediated modulation of the secretion of gut hormones^26^. The higher abundance of *Firmicutes* and *Bacteroidetes* in yak may be associated with high-energy consumption at high altitude^18^.

It is worth noting that, at the family level, the dominant genera (*Unclassified Ruminococcaceae*, *Bacteroidaceae, Unclassified BS11*, *Unclassified Prevotellaceae*, *Unclassified Christensenellaceae*, *CF231*, *Unclassified Mogibacteriaceae* and *Unclassified Paraprevotellaceae)* in the intestines of yak and Tibetan sheep were more greatly influenced by season than genetics (Fig. 5). This has not previously been accurately identified, which may be because there have been few studies into the gut microbial communities of the yak and Tibetan sheep in QTP. So, to improve their husbandry, it is important in the future to study their microbiota profiles using more precise methods such as 16S full-length sequencing or metagenomic sequencing. *Ruminococcaceae* is a family of autochthonous and benignspecies that primarily inhabit in the caecum and the colon^27^. It is known that Ruminococcaceae are common in the rumen and hindgut of ruminants, capable of degrading cellulose and starch^28^. As a member of short chain fatty acid (SCFA) producers, *Ruminococcaceae* is considered to be the most important fiber and polysaccharides-degrading bacterium in the intestine of herbivores, and produces large amounts of cellulolytic enzymes, including exoglucanases, endoglucanase, glucosidases and hemicellulase^29^. The microbial community of Yak and Sheep is greatly influenced by alterations in dietary nutrition, *Bacteroidaceae* have the ability to degrade complex molecules (polysaccharides, proteins) in the intestine^18^, which can promote the Yak utilizes grasses as its major source of nutrition, due to shortage of grain and other nutrients. *Prevotellaceae* is responsible for hemicellulose, pectin and high carbohydrate food digestion^30^. The higher abundance of these microbes may contribute to gaining more energy, and play vital roles in the process of adaption of the hosts to the harsh natural environment^15^. *Bacteroidales BS11* gut group are specialized to active hemicellulose monomeric sugars (e.g., xylose, fucose, mannose and rhamnose) fermentation and short-chain fatty acid (e.g., acetate and butyrate) production that are vital for ruminant energy^31^. The *Bacteroidales BS11* was positively correlated with some metabolites that are involved in amino acid metabolism and biosynthesis, as well as the metabolism of energy sources, such as starch, sucrose, and galactose^32^.

At the genus level, *5-7N15* was most abundant in winter in both animals, on the contrary, the *Provotella* was predominate. Here, our results indicated that seasonal diets change superseded variations derived from genetic differences between the host species, even though the yak and Tibetan sheep are very different, both taxonomically and in terms of body size. In summer, the forage grass on the Qinghai-Tibet Plateau is dominated by *Agropyron cristatum, Elymus nutans, Festuca ovina, Kobresia humilis, Poa pratensis, Stipa aliena, Kobresia pygmaea, Oxytropis biflora, Saussurea hieracioides, Astragalus arnoldii Hemsl*. In winter, the main forage was *Brachypodium sylvaticum. Carex crebra, Trisetum spicatum* and *Bupleurum smithii.* Stipa has both high palatability and nutritional value, with a high content of crude protein, crude fat, and nitrogen- free extract, and low levels of crude fiber^33^. The levels of crude protein, crude fat, and nitrogen-free extracts of *Brachypodium sylvaticum. Carex crebra, Trisetum spicatum* and *Bupleurum smithii* were lower than that of Stipa, whereas the content of crude fiber was higher than that of Stipa^34^. Crude protein is the main nutrient of herbage. Crude fat and nitrogen-free extracts provide heat and energy^33^.

Lopes et al. reported that some OTUs known to be functionally relevant for fiber degradation and host development were shared across the entire gastrointestinal tract and present within the feces^35^. Microbial diversity increases in the distal segments of the gastrointestinal tract. Microbial fermentation appears to be reestablished in the large intestine, with the proportion of acetate, propionate and butyrate being similar to the rumen.

Several explanations for this phenomenon are possible. Firstly, both the yak and Tibetan sheep are ruminants. In herbivores, the gut microbiota is dominated by Firmicutes and Bacteroides, the functions of which are related to cellulose digestion^36^. Therefore, ruminant microbes could possibly be more similar across species than gut microbes from elsewhere.

Secondly, the yaks and Tibetan sheep in our study co-grazed from birth to death. As such, the initial gut microbiota source, responsible for populating the remainder of the gut in the months and years after the initial seeding at birth, would necessarily come from the same environment. It has been established that early life events are critical for gut microbiota development and for shaping the adult microbiota. Lifestyle and diet will further influence the composition and function of the gut microbiota. In our study, the investigated animals shared a very similar lifestyle and obtained their diets from the same source. The results revealed that sheep and yaks presented almost identical gut microbiota compositions in the winter, but by the date of collection of the summer samples they were quite different. The reason for this could be that during summer and summer there is pronounced pastoral grass growth, giving the animals more variety and choice in their diets; it is known, after all, that sheep have different diet preferences to yaks^37^. However, during the winter, the animals have no option but to eat the same food in order to survive until winter.

Thirdly, there could be a convergent evolution of gut microbiomes in yaks and Tibetan sheep due to the extremely harsh environment in high-altitude regions^1,38^. When compared with their low-altitude relatives, cattle (*Bos taurus*) and ordinary sheep (*Ovis aries*), metagenomic analyses revealed significant enrichment in rumen microbial genes involving volatile fatty acid-yielding pathways in yaks and Tibetan sheep, whereas methanogenesis pathways were enriched in the cattle metagenome. Analyses of RNA transcriptomes revealed significant upregulation in 36 genes associated with volatile fatty acid transport and absorption in the ruminal epithelium of yaks and Tibetan sheep. This suggests that, aside from host genetics, long-term exposure to harsh environments has allowed the gut microbiome to adapt in order to boost health and survival. In other words, although yaks and Tibetan sheep are very different genetically, their gut microbiota could be similar due to the selection pressures of the high altitude at which they live. Meanwhile, from our data based on functional gene composition (Fig. S3), it is also worth noting that there were no groups clearly distinguished from one another, although the PERMANOVA results indicated both a host and season effect, with the interaction between them being statistically significant. Though factors such as environment and diet (represented by seasons) can trump host genetics, we could not ignore the interplay of these factors as gut microbes are a very complex community.

Winter is the harshest period for the survival of yak and Tibetan sheep. To maintain the survival, it’s best to feed the animals with a high protein content. Furthermore, to get more detailed data in different seasons and various dietary habits of yak and sheep, more study should be assessed about intestinal microbiota by collecting feces.

## Methods

### Study site and sampling

To investigate the influence of hosts and seasons on the gut microbiome diversity component, we collected fecal samples from yaks and Tibetan sheep across different seasons (summer and winter), during which they consumed different diets. The study area was located at Oula Village of the Maqu Wetland Protection Area (E 100°45′–102°29′, N 33°06′–34°30′) in Gansu Province, China. The village is situated in the eastern part of the QTP with an average altitude > 3000 m above sea level. The mean daily air temperature was 1.2 °C, with the lowest mean air temperature reaching − 10 °C in January and highest reaching 11.7 °C in July. The mean annual precipitation was 620 mm, with the majority falling during the summer. The grazing pastures for the animals consisted of typical alpine meadows, containing mainly the following vegetative species: *Kobresia kansuensis, Thalictrum aquilegifolium var. sihiricum, Stipa capillata, Potentilla fragarioides, Saussurea hieracioides, Taraxacum mongolicum, Anemone baicalensis var. kansuensis, Anemone rivularis var. flore-minore, Euphorbia esula, Medicago ruthenica,* and *Plantago asiatica*. Yak and Tibetan sheep were co-grazed, alongside some wild animals, such as *prairie dog*, *wild ass and Sika Deer* without any supplementary feeding. During the study, the yak population was 232, ranging in age between 1–3 years; there were also 300 Tibetan sheep aged 1–1.5 years of age.

Sampling procedures were performed twice. Winter season samples were collected on March 29, 2016, after melting of the snow and before sprouting of the grass. Summer samples were collected on September 3, 2016, at approximately the point when animals were at their highest body condition score. Sampling was performed in the early morning. We followed behind the yaks; fecal samples were collected immediately after spontaneous defecation using a sterile spatula. When sampling, the surface layers was removed and collected from the center of excrement to avoid contamination. The resultant stool samples were stored in a centrifuge tube and kept in liquid nitrogen until extraction of genomic DNA. 136 yak fecal samples were collected (56 from winter, thereafter coded as WinY; 80 from summer coded as SumY). 90 Tibetan sheep fecal samples were collected (43 from winter coded as WinS and 47 from summer coded as SumS). In total, 226 fresh fecal samples were collected.

All of the experimental protocols and procedures were approved by the Institutional Animal Care and Use Committee of Lanzhou Institute of Husbandry and Pharmaceutical Science of the Chinese Academy of Agricultural Sciences. Animal welfare and experimental procedures were performed strictly in accordance with the Guidelines for the Care and Use of Laboratory Animals issued by the US National Institutes of Health.

### DNA extraction, PCR amplification, and high-throughput sequencing

Genomic DNA was extracted from fecal samples using a TIANamp Stool DNA Kit (TIANGEN, Beijing, China.) according to the manufacturer’s instructions. The quality and quantity of extracted DNA were assessed using a NanoDrop ND 1000 spectrophotometer (NanoDrop Technologies, USA). The V3–V4 region of the 16S rRNA gene was amplified using barcoded primers. Amplicons were extracted from 2% agarose gels and purified using an DNA Gel Extraction Kit (AxyPrep, Hangzhou, China) according to the manufacturer’s instructions and quantified using QuantiFluor (Promega, USA). Purified amplicons were pooled in equimolar and paired-end sequences (2 × 300 bp) using an Illumina MiSeq platform according to standard protocols. Sequencing procedures were delegated to a commercial company: Shanghai Personalbio Technology Co., Ltd.

### Data analysis

Operational taxonomic units (OTUs) with a 97% similarity cutoff were clustered using UPARSE (version 7.1)^39^, and chimeric sequences were removed using UCHIME^40^. The taxonomy of each 16S rRNA gene sequence was analyzed against the Greengenes database^41^ at a confidence threshold of 70%. Rarefaction analysis based on Mothur v.1.35.1 (https://www.mothur.org). ^42^ was conducted to reveal diversity indices, including ACE, Chao1, Shannon, Simpson, and coverage indices^43–46^. Two-way ANOVA was utilized to explore the effects of season and host species on the richness, evenness, and diversity of microbial communities. Beta diversity of the Bray–Curtis distance between the samples in the same groups and between different groups were analyzed, with box-plots generated to show differences. Principal coordinate analysis (PCoA) plots were generated to compare bacterial/archaeal community composition among samples from different conditions. Permutational multivariate analysis of variance (PERMANOVA) was performed on the Bray–Curtis metric produced by PCoA analysis to test for significant differences in community composition among the treatments. All the above analyses were completed using R software (versions 3.3.3)^47^. Non-parametric ANOVA analysis was conducted using the “ImPerm” package; multivariate analyses were conducted with the “vegan” package. Phylogenetic Investigation of Communities by Reconstruction of Unobserved States (PICRUSt) was used to predict metagenome functional content from 16S rRNA gene surveys (http://picrust.github.com). ^48^. Venn diagrams were constructed to show unique or shared OTUs and also KEGG^49,50^ functional genes predicted by PICRUSt.

## Supplementary Information

Supplementary Figure S1.

Supplementary Figure S2.

Supplementary Figure S3.

Supplementary Information.

## Data Availability

The datasets generated for this study can be found in NCBI GenBank, accession numbers are SRP148671 (https://www.ncbi.nlm.nih.gov/sra/SRP148671) and SRP148670 (https://www.ncbi.nlm.nih.gov/sra/SRP148670).
