# Supplementary figures and images for: Seasonal diets supersede host species in shaping the distal gut microbiota of Yaks and Tibetan sheep
Source: Sci Rep. 2021 Nov 19;11:22626. doi: 10.1038/s41598-021-99351-4 (PMC8604981; doi:10.1038/s41598-021-99351-4)

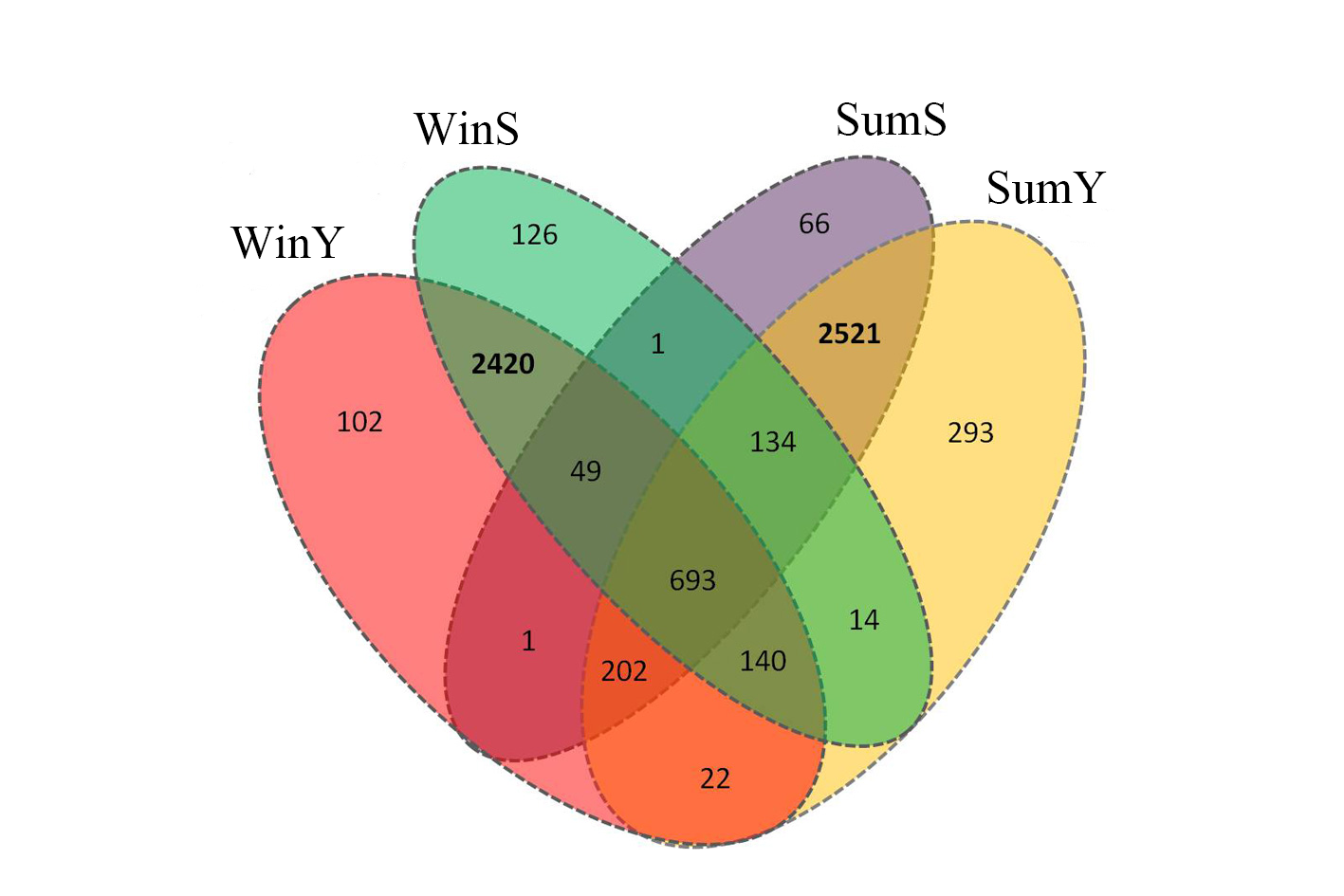

Supplement: Supplementary file 1 — Supplementary Figure S1. [file 41598_2021_99351_MOESM1_ESM.tif]

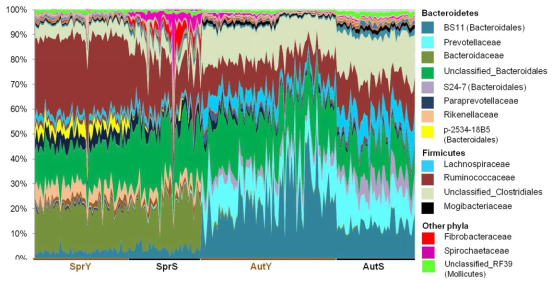

Supplement: Supplementary file 2 — Supplementary Figure S2. [file 41598_2021_99351_MOESM2_ESM.tif]

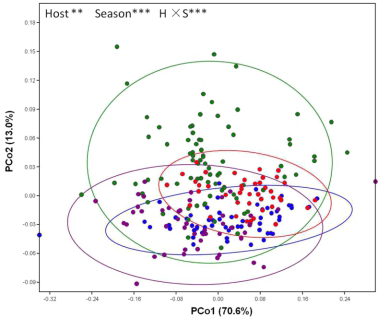

Supplement: Supplementary file 3 — Supplementary Figure S3. [file 41598_2021_99351_MOESM3_ESM.tif]
