# Supplementary material for: Seasonal diets supersede host species in shaping the distal gut microbiota of Yaks and Tibetan sheep
Source: Sci Rep. 2021 Nov 19;11:22626. doi: 10.1038/s41598-021-99351-4 (PMC8604981; doi:10.1038/s41598-021-99351-4)
Supplement: Supplementary file 4 — Supplementary Information. [file 41598_2021_99351_MOESM4_ESM.docx]

figures file

Figure S1. Venn diagrams of the operational taxonomic units (OTUs).

Figure S2. Prokaryotic community composition at the family level. The y-axis shows the values of the relative abundances of families. The x-axis shows the samples which were grouped by host and season.

Figure S3. Principal coordinates analysis (PCoA) ordination of the KEGG functional genes predicted by PICRUSt. Dots indicate one sample and the circles are the 95% ellipses. Colors are as follows: blue = WinY; red = WinS; green = SumY; purple = SumS. Results of PERMANOVA are given in the upper right of each panel: **, P < 0.01; ***, P < 0.001.
